# Supplementary material for: Chronic kidney disease causes and outcomes in children: Perspective from a LMIC setting
Source: PLoS One. 2022 Jun 8;17(6):e0269632. doi: 10.1371/journal.pone.0269632 (PMC9176774; doi:10.1371/journal.pone.0269632)
Supplement: Attachment — Submitted filename: Response Letter_20220301.docx [file pone.0269632.s002.docx]

March 1, 2022

Dear Dr. Bhimma

We would like to thank you and the reviewers for your careful review of this manuscript and the insightful comments to help improve our work. We have attempted to address all the concerns that have been raised. We believe that the manuscript is stronger as a result. Please see our point-by-point responses in red below.

As this is review of medical records for which requirement for informed consent was waived, data can be accessed upon email to the corresponding author and the Institutional Review Board (IRB) of IRD at [irb@ird.global](mailto:irb@ird.global).

On behalf of the authorship team,

Amyn Malik

**Comments to the Author**
Reviewer #1: Thank you for the opportunity to review this interesting paper.
The paper is a retrospective review of 229 children who presented in various stages of CKD.
The introduction adequately sets the background for the study and the method used is appropriate for the report. The findings are in keeping with previously published work and highlight the importance of early presentation to prognosis. The discussion contextualises the results well and the conclusions are inciteful.

Authors: We appreciate your insightful comments on our work and thank you for your time to review it.

I would suggest that the paper be accepted for publication but that a few minor changes are made as follows:
The Introduction
• The statement starting on Line 71 needs referencing

Authors: Reference to the online registry maintained by International Pediatric Nephrology Association (IPNA) has been added.

• The statement starting on Line 75 is unclear as to its meaning and needs to be rephrased and referenced

Authors: We have rephrased and referenced this statement as follows:

“Pakistan ranked 7th highest in terms of number of children on hemodialysis.(9)”

The Discussion

• The sentence beginning on Line 233 does not read well and is too long. It should be rephrased

Authors: Thank you for the suggestion. We have rephrased this as follows:

“An evaluation of overall outcomes showed that only 46% children (82% of whom had non-glomerular CKD) in our cohort were doing well in stable follow-up. More than half the children had poorer renal and possibly survival outcomes, highlighting the terminal nature of childhood ESRD in our setting where services are limited and are not available closer to patient’s homes.”

• The sentence starting on Line 242 does not read well and needs to be rephrased

Authors: We have rephreased this sentence as follows:

“An important finding of our study is that in a pediatric urology referral center such as ours, prompt referral, diagnosis and integrated management (including urologic surgery) may have helped improve outcomes in the non-glomerular/ urologic CKD group.”

• The sentence starting on Line 266 has a full stop in error between the word “cohort” and the word “which” (Line 267) and “which” should be spelt with a small case w

Authors: Thank you for pointing this out. We have corrected this error.

The Tables
• I am not sure that the title of Table 5 is correct. I think that the features mentioned would be regarded as manifestations of, rather than risk factors for, late-stage CKD

Authors: Thank you for the suggestion. We agree that these are associations/manifestations of late-stage CKD. We have corrected the title accordingly.

Reviewer #2: The study is relevant and informative. A letter from the institution allowing the use of anonymized data would strengthen the ethical standards required for the paper. There is a valid rationale for the study. Research questions are plainly identified and acceptable but the research question should mention a comparison between early and late stage chronic kidney disease as well as a comparison of glomerular and non-glomerular disease. These important comparisons are provided in the results.

Authors: Thank you for these suggestions. We have added the following to the introduction:

“This study is designed to assess demographics, underlying cause of CKD, clinical stage at presentation, and the association of the underlying cause and stage with clinical outcomes among children registered in the pediatric CKD clinic at The Indus Hospital (TIH) in Karachi, where all services are provided free of cost.”

We have also uploaded a separate IRB waiver letter in the system as well.

The protocol was technically sound and well planned. The outcome was valid and the stated hypothesis was adequately tested. However, results from the analysis are mentioned under discussion and should be fully reassigned to the results section before they can be discussed later. The discussion should also make methodical reference to the results obtained.

Authors: Thank you for pointing this out. We have moved all results from the discussion section to the results section and have referenced the discussion accordingly with out results.

The data is available for scrutiny. The methodology is feasible and can likely be reproduced and replicated. The statistical analysis appear valid. Overall an insightful study that adds to the existing body of knowledge.

Authors: We appreciate your insightful comments on our work and thank you for your time to review it.
