# Supplementary material for: Chronic kidney disease causes and outcomes in children: Perspective from a LMIC setting
Source: PLoS One. 2022 Jun 8;17(6):e0269632. doi: 10.1371/journal.pone.0269632 (PMC9176774; doi:10.1371/journal.pone.0269632)
Supplement: Attachment — Submitted filename: Response to Reviewers.docx [file pone.0269632.s003.docx]

April 25, 2022

Dear Dr. McDonald

We would like to thank you and the reviewers for your careful review of the revised manuscript to help improve our work. We have attempted to address all the remaining concerns. We believe that the manuscript is stronger as a result. Please see our point-by-point responses in red below.

On behalf of the authorship team,

Amyn Malik

**Comments to the Author**
Reviewer #1: The paper looks great. The only typo left to correct is the reference in the sentence starting on Line 71 'According to the International Pediatric Nephrology Association’s (IPNA)(8) Renal Replacement Therapy Registry......' should be moved to after the word 'centres' on line 73 as follows: "According to the International Pediatric Nephrology Association’s (IPNA) Renal Replacement Therapy Registry, of the 23000 children on chronic dialysis in 2018, 246 children were registered dialysis patients from Pakistani centers.(8)

Authors: Thank you. We have corrected the reference placement as advised.

Reviewer #2: Kindly add the anaemia prevalence results on line 238 and 239 to the results section and add the corresponding discussion around anaemia in the discussion.

The rest of the paper is readily acceptable.

Authors: Thank you. We have added the anemia results and the relevant discussion as follows:

“Similarly, children with CKD stages 3-5 had an anemia prevalence of 81% (n=48/64) compared to 33% (n=52/165) in the earlier CKD group (Table 5). Presence of proteinuria, hypertension, anemia, and mineral bone disorder were statistically significantly associated with adverse outcomes.” (line 181-184)

“We found an anemia prevalence of 81% in our late CKD cohort (compared to 33% in the earlier CKD group) and a significant association with dialysis and death. NAPRTCs reports an anemia prevalence of 73% at stage 3 CKD, 87% at stage 4 and >93% at stage 5.(29) Anemia is known to be a risk factor for poor outcomes including poor growth, neurocognitive problems and is an independent predictor of mortality in adolescents on hemodialysis.(30, 31)” (line 239-244)
